# The immune landscape of the microenvironment of thyroid cancer is closely related to differentiation status

**DOI:** 10.1186/s12935-021-02084-7

**Published:** 2021-07-20

**Authors:** Lucas Leite Cunha, Guilherme Augusto Barcelos Domingues, Elaine Cristina Morari, Fernando Augusto Soares, José Vassallo, Laura Sterian Ward

**Affiliations:** 1 Laboratory of Molecular and Translational Endocrinology, Division of Endocrinology, Federal University of São Paulo, 669, Pedro de Toledo St, 11st floor, São Paulo, 04039-032 Brazil; 2 Laboratory of Cancer Molecular Genetics, Faculty of Medical Sciences, University of Campinas (Unicamp), Campinas, Brazil; 3 Federal University of Roraima, Boa Vista, Brazil; 4 Pathology Division, ID’ Or Research Institute, Rede D’Or Hospitals Network, São Paulo, Brazil; 5 Laboratory of Investigative and Molecular Pathology (Ciped), Faculty of Medical Sciences, University of Campinas (Unicamp), Campinas, Brazil

**Keywords:** Thyroid cancer, Immune microenvironment, Tumor dedifferentiation

## Abstract

We have read with great interest the article entitled “Identification of an immune-related signature indicating the dedifferentiation of thyroid cells” by Wang et al. Their data reinforce our own previous results, here compiled. Anaplastic thyroid carcinoma had higher stromal scores, immune scores and enrichment of most immune cells than the control groups, suggesting that the immune microenvironment may correlate with differentiation status in thyroid cancer. We previously demonstrated that the differentiation status expressed by the pattern of protein expression may be related to the profile of immune cell infiltration of differentiated thyroid carcinoma. Wang et al. also explored the differences between the high-risk and low-risk score groups of samples. Among the distinct signaling pathways enriched in the high-risk score group, the epithelial to mesenchymal transition, TNFα signaling, and some common immune-related signaling pathways, including the IL-6/JAK/STAT3 pathway, interferon alpha response, interferon gamma response and inflammatory response were observed with high normalized enrichment score. We also investigated the IL-6 protein immune-histochemical expression in a retrospective study of 114 patients with papillary thyroid carcinoma and 39 patients with follicular thyroid carcinoma. We also obtained samples of 14 normal thyroid tissues from autopsies, 50 goiters and 43 follicular adenoma. We found IL-6 more frequently positive among malignant tumors than non-malignant samples. We demonstrated that IL-6 positivity was associated with infiltration of CD3 + cells, CD16 + cells and CD68 + macrophages. In addition, IL-6 expression was associated with infiltration of activated lymphocytes such as Granzyme B + cells and CD69 + cells. IL-6 positivity was not associated with infiltration of CD4+, CD8+, CD20+, FOXP3+, CD25 + cells but IL-6 was associated with tumor expression of PD-L1, FOXP3, IL-17, COX2, IL-1β, IL-10, CD134, IL-23. In summary, Wang et al. beautiful data reinforce the seminal idea that the immune landscape is closely related to the differentiation status of the tumor. This concept may help select individuals who deserve more careful attention, an essential point in the management of patients with mostly indolent tumors such as those of the thyroid. In fact, our results, here compiled, were obtained with immune-histochemistry, a routine laboratory technique that offers the possibility of simpler and practical execution.

## Background

We have read with great interest the article entitled “Identification of an immune-related signature indicating the dedifferentiation of thyroid cells” by Wang et al. [1]. Their data reinforce our own previous results, here compiled.

Wang et al. performed a carefully molecular and *in silico* investigation of the immune landscape of the microenvironment of thyroid cancer. They systematically searched for publicly available anaplastic thyroid carcinoma (ATC) transcriptome datasets and used transcriptome data and clinical information of papillary thyroid carcinomas (PTCs) from the TCGA database downloaded from the UCSC Xena browser in order to perform further comparisons. A list of 16 thyroid differentiation score (TDS) genes was obtained from a published study investigating PTC and served as a parameter of thyroid differentiation enabling a comparison among the different samples. As expected, ATC presented lower TDS. The stromal score and immune score of each sample were calculated by the ESTIMATE package in the R program. ATC had higher stromal scores, immune scores and enrichment of most immune cells than the control groups, suggesting that the immune microenvironment may correlate with differentiation status in thyroid cancer.

## Main text

We previously demonstrated that the differentiation status expressed by the pattern of protein expression may be related to the profile of immune cell infiltration of differentiated thyroid carcinoma [2]. It is possible that the antigenicity of thyroid cancer cells may facilitate the inflammatory milieu that condition the migration of immune cells to thyroid microenvironment. According to this hypothesis, the differentiation status may be a result of the molecular profile that leads both to a change in the inflammatory microenvironment and to the clinical phenotype.

In order to further analyze the differentiation-associated immune-related genes, Wang et al. enrolled 505 PTC samples with complete clinical annotations and transcriptome data from TCGA database. These cases were then divided into a low-differentiated group and a high-differentiated phenotype. The authors observed that both the interferon gamma and the inflammatory responses were significantly enriched in the low-differentiated group, supporting the idea that differentiation and inflammation cannot be dissociated.

The authors further constructed a model of two-genes (MMP9, matrix metalloproteinase-9 and SDC2, syndecan-2) risk score and observed that this model was closely correlated with the immune-related signatures. They found that the expression of MMP9 in ATCs and low differentiated PTCs was higher than that in normal tissues and high-differentiated PTCs. The SDC2 displayed the opposite expression profile. In addition, an immune-related risk score was constructed and was obviously higher in ATCs and low-differentiated PTCs. The correlation analyses proved that the risk score was negatively associated with differentiation.

Expanding their investigation, Wang et al. observed a markedly positive correlation between the risk score and important immune checkpoint molecules, including programmed cell death ligand 1 (PD-L1). Moreover, they found that most immune cells displayed higher enrichment score in the high-risk score group than the low-risk score group. The inflammation that occurs at tumor level may be an epiphenomenon driven by the set of molecular alterations that occurs to the thyroid cancer cells. In fact, inflammatory microenvironment may be protective or, on the contrary, deleterious to the host depending on how the interaction with the thyroid cancer cells is processed [3]. It is also possible that the production of co-inhibitory molecules by cancer cells, such as PD-L1, may predispose the tumor immune microenvironment to a pro-cancer inflammation [4], leading to a more aggressive clinical presentation. We previously assessed the expression of PD-L1 and immune landscape of the microenvironment of 407 patients whose tissue samples were maintained in our tissue bank [5]. A total of 293 patients were diagnosed with thyroid carcinoma, including 253 with PTC (153 cases of the classical form; 80 follicular variants; and 20 tall-cell variants) and 40 with follicular thyroid carcinomas (22 minimally invasive and 18 frankly invasive). We compared immune microenvironment with non-malignant tissues obtained from 5 normal and 114 benign thyroid tissues, including 58 nodular goiters and 56 follicular adenomas. We demonstrated that malignant tissues had a more intense PD-L1 staining than benign tissues; PTC samples presented more intense staining than goiters; and poorly differentiated variants presented the higher expression of PD-L1. In addition, we observed a positive correlation between upregulated PD-L1 mRNA levels and the presence of CD3 + lymphocytes, CD4 + lymphocytes, CD8 + lymphocytes, and FOXP3 + lymphocytes, reinforcing that PD-L1 expression by thyroid cancer cell is intrinsically correlated with the profile of immune microenvironment [5].

**Fig. 1:**
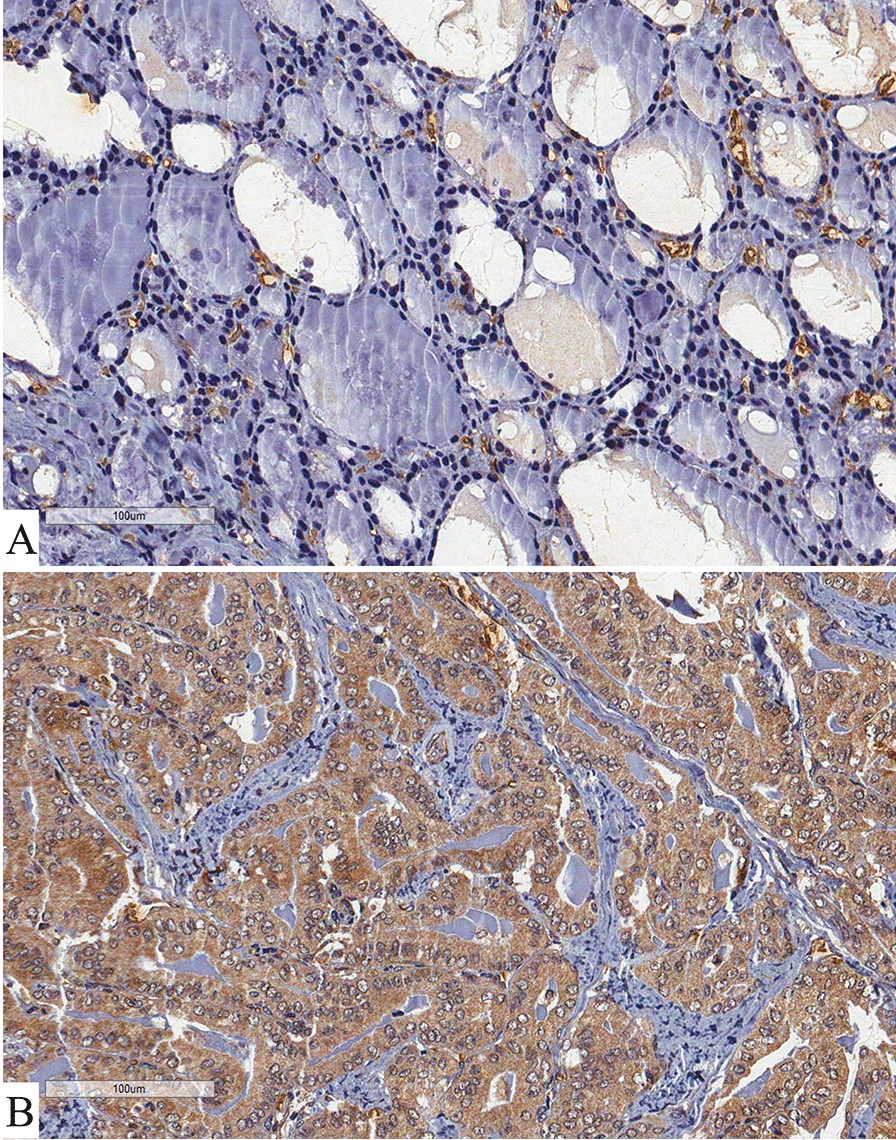
Immunohistochemistry of IL-6. Panel ** A** shows the faint diffuse brownish in cytoplasm of thyroid goiter cells (benign tissue). Panel ** B** evidence that major cytoplasmic intense diffuse brownish of thyroid cancer cells in a classic papillary thyroid carcinoma (malignant tissue)

Wang et al. also explored the differences between the high-risk and low-risk score groups of samples. Among the distinct signaling pathways enriched in the high-risk score group, the epithelial to mesenchymal transition, TNFα signaling, and some common immune-related signaling pathways, including the IL-6/JAK/STAT3 pathway, interferon alpha response, interferon gamma response and inflammatory response were observed with high normalized enrichment score. We also investigated the IL-6 protein immune-histochemical expression in a retrospective study of 114 patients with PTC (60 with classic PTC, 44 with follicular variant of PTC and 10 with poorly differentiated histologic phenotype of PTC) and 39 patients with follicular thyroid carcinoma. We obtained samples of 14 normal thyroid tissues from autopsies, 50 goiters and 43 follicular adenoma. We classified as poorly differentiated PTC tumors those with solid, trabecular or insular growth patterns, with no nuclear features of PTC and with one of the following features: convoluted nuclei, 3 or more mitotic figures per 10 high power field, and tumor necrosis [6]. We also evaluated the presence/absence of both microscopic and gross extrathyroidal invasion and staged our patients according to the AJCC 8th edition criteria of aggressiveness at diagnosis [7]. All patients were carefully followed-up by a previously described protocol for a period of 1-242 months (mean 57.5 ± 41.1 months) [8]. Immunohistochemistry, defined by a clear cut brown staining observed in the corresponding cellular localization, clearly distinguished positivity, as exemplified by Fig. 1.

In addition, semiquantification was performed by two investigators (LLC and ECM), as routinely done by our group [8, 9]. We found IL-6 more frequently positive among malignant tumors (66.7 %) than non-malignant samples (41.1 %; p < 0.05). In fact, IL-6 was positive in 71.7 % of classic PTC, 54.5 % of follicular variant of PTC, 90.0 % of poorly differentiated PTC and 66.7 % of follicular thyroid carcinoma (p = 0.11). None of the normal thyroid tissues was positive for IL-6. Only 34.0 % of the goiters were positive for IL-6 and 62.8 % of follicular adenomas were positive for IL-6, suggesting that the positivity for IL-6 increases according to the further steps that thyroid cells reach towards malignant transformation. The higher expression of IL-6 among poorly differentiated neoplasms reinforce Wang et al. proposition that the IL-6/JAK/STAT3 pathway may impact risk-score in thyroid cancer. IL-6 expression was not associated with presence/absence of metastasis at diagnosis, staging and relapse-free survival (all p > 0.05). We demonstrated that IL-6 positivity was associated with infiltration of CD3 + cells (p = 0.013), CD16 + cells (p < 0.001) and CD68 + macrophages (p = 0.001). In addition, IL-6 expression was associated with infiltration of activated lymphocytes such as Granzyme B + cells (p = 0.002) and CD69 + cells (p < 0.001). IL-6 positivity was not associated with infiltration of CD4+, CD8+, CD20+, FOXP3+, CD25 + cells (p > 0.05) but IL-6 was associated with tumor expression of PD-L1 (p < 0.001), FOXP3 (p < 0.001), IL-17 (p = 0.004), COX2 (p < 0.001), IL-1β (p < 0.001), IL-10 (p < 0.001), CD134 (p < 0.001), IL-23 (p < 0.001).

## Conclusions

**Fig. 2:**
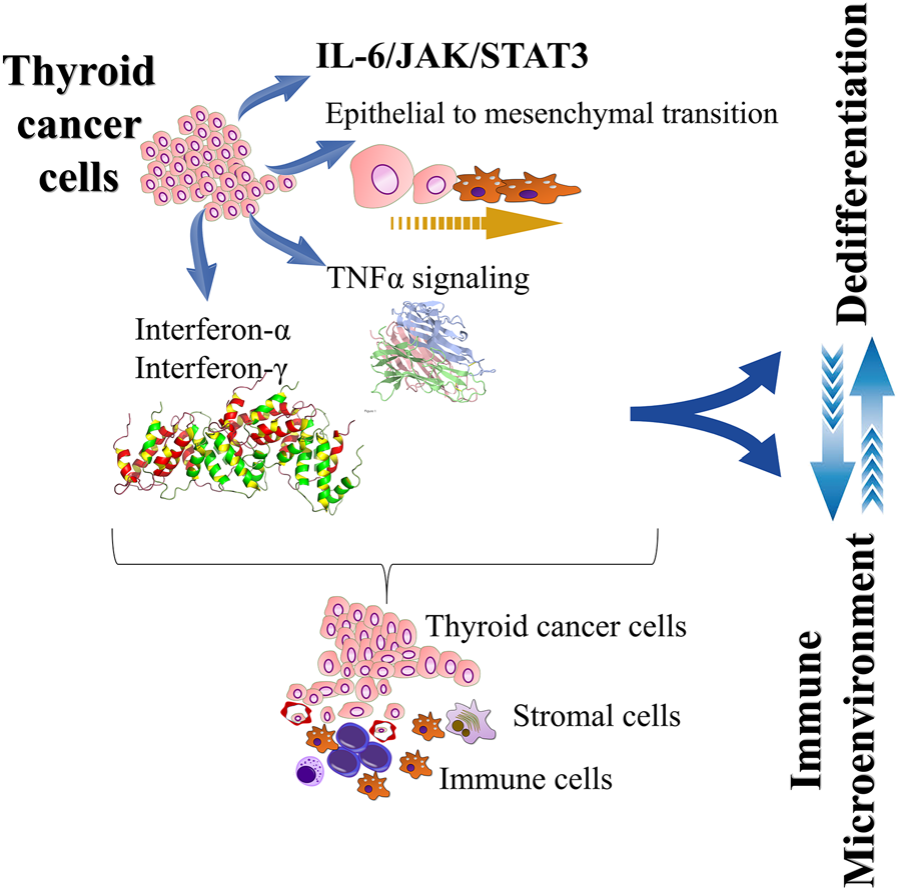
Schematic panel evidencing the complexity of dedifferentiation status and inflammatory microenvironment of thyroid cancer. As demonstrated by Wang et al., thyroid carcinoma cells can exhibit high-risk dedifferentiation behavior, which enhances the epithelial to mesenchymal transition, TNFα signaling, and some common immune system-related signaling pathways, including the pathway IL-6 / JAK / STAT3, interferon alpha and gamma response. Furthermore, the dedifferentiation status appears to be accompanied by an immunological profile characteristic of the immune microenvironment of the tumor, suggesting that the two are closely related

In summary, Wang et al. beautiful data reinforce the seminal idea that the immune landscape is closely related to the differentiation status of the tumor, as outlined in Fig.  2. This concept may help select individuals who deserve more careful attention, an essential point in the management of patients with mostly indolent tumors such as those of the thyroid. In fact, our results, here compiled, were obtained with immune-histochemistry, a routine laboratory technique that offers the possibility of simpler and practical execution.

## Data Availability

Data sharing is not applicable to this article as no datasets were generated or analysed during the current study.
